# Human microRNAs in host–parasite interaction: a review

**DOI:** 10.1007/s13205-020-02498-6

**Published:** 2020-11-05

**Authors:** Sujay Paul, Luis M. Ruiz-Manriquez, Francisco I. Serrano-Cano, Carolina Estrada-Meza, Karla A. Solorio-Diaz, Aashish Srivastava

**Affiliations:** 1 Tecnologico de Monterrey, School of Engineering and Sciences, Campus Queretaro, Av. Epigmenio Gonzalez, No. 500 Fracc. San Pablo, 76130 Querétaro, Mexico; 2 Section of Bioinformatics, Clinical Laboratory, Haukeland University Hospital, 5021 Bergen, Norway; 3 Department of Clinical Science, University of Bergen, 5021 Bergen, Norway

**Keywords:** microRNAs, Human parasitic diseases, Pathophysiology, Prognosis, Biomarker

## Abstract

MicroRNAs (miRNAs) are a group of small noncoding RNA molecules with significant capacity to regulate the gene expression at the post-transcriptional level in a sequence-specific manner either through translation repression or mRNA degradation triggering a fine-tuning biological impact. They have been implicated in several processes, including cell growth and development, signal transduction, cell proliferation and differentiation, metabolism, apoptosis, inflammation, and immune response modulation. However, over the last few years, extensive studies have shown the relevance of miRNAs in human pathophysiology. Common human parasitic diseases, such as Malaria, Leishmaniasis, Amoebiasis, Chagas disease, Schistosomiasis, Toxoplasmosis, Cryptosporidiosis, Clonorchiasis, and Echinococcosis are the leading cause of death worldwide. Thus, identifying and characterizing parasite-specific miRNAs and their host targets, as well as host-related miRNAs, are important for a deeper understanding of the pathophysiology of parasite-specific diseases at the molecular level. In this review, we have demonstrated the impact of human microRNAs during host−parasite interaction as well as their potential to be used for diagnosis and prognosis purposes.

## Introduction

**Fig. 1:**
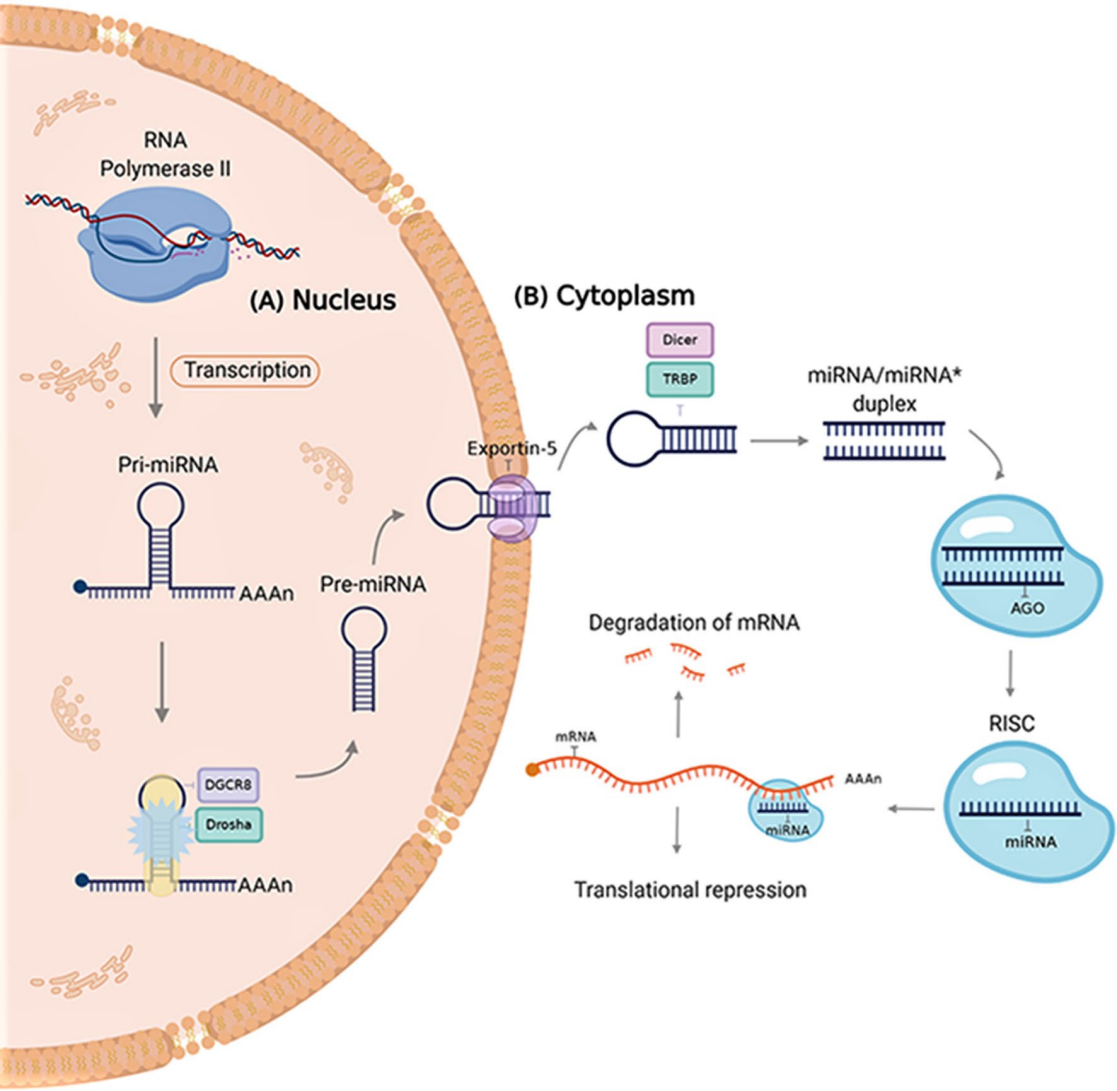
The canonical pathway of miRNA biogenesis. **a** In the nucleus, the miRNA gene is transcribed by RNA polymerase II and then fold into a long pri-miRNA with a hairpin structure. The long pri-miRNA is then cleaved by the microprocessor complex made up of Drosha and DGCR8 (Pasha) proteins, generating a precursor miRNA (pre-miRNA). **b** Exportin-5 binds to the pre-miRNA and facilitates its export to the cytoplasm. In the cytoplasm, the type III RNase Dicer complex with the double-stranded RNA binding protein TRBP and PACT cleaves the precursor’s hairpin and the resulting duplex is isolated by a helicase enzyme. Finally, the functional strand is loaded together with the Argonaute (AGO) protein into the RNA-induced silencing complex (RISC) to target mRNAs by sequence complementary binding and mediates gene suppression through mechanisms of either translational repression or mRNA degradation

miRNAs are small (~ 22 nucleotides in length), endogenous, evolutionarily conserved regulatory ncRNAs that are implicated in the post-transcriptional regulation of cellular signaling pathways in both animals and plants (Paul et al. 2018, 2020a, d; De la Fuente et al. 2020). Because the discovery of these molecules in *Caenorhabditis elegans* by Lee et al. (1993) it has been shown that they are widely distributed in most eukaryotes, including humans (Felden and Gilot 2019; Paul et al. 2020b, c). Biogenesis of miRNAs consists of sequential events occurring in the cell nucleus and cytoplasm. In the nucleus, miRNA genes are first transcribed by the RNA polymerase II and fold into long double-strand primary miRNA transcripts (pri-miRNA). Then, the RNase type III Drosha and DGCR8 (also known as Pasha) complex processes the pri-miRNA molecule to form miRNA precursor (pre-miRNA) (Fig. 1a). The resulted pre-miRNA is then translocated into the cytoplasm by Exportin 5 where it is processed one more time by the complex composed of the RNase III Dicer and the Trans-Activation Responsive RNA-Binding Protein (TRBP) to form the mature miRNA/miRNA* duplex (Treiber et al. 2019). To be functional, the resulted miRNA/miRNA* duplex is then separated by a helicase and the single miRNA strand is incorporated into the RNA-induced silencing complex (RISC) coupled with the Argonaute (AGO) protein family which guides to interact with the target mRNA. Binding of miRNAs to the mRNAs (partial or full complementarity) leads to the regulation of their expression either by the degradation of the mRNA or by inhibiting its translation (Fig. 1b) (Wang 2014).

Evidence has shown that miRNAs influence numerous cell biological processes including growth and development, signal transduction, cell proliferation and differentiation, metabolism, cell death, and immune regulation (Miska 2005; Wu and Lu 2017; Pockar et al. 2019). It has been reported that miRNAs participate in the regulation of nearly 60% of human protein-coding genes (Friedman et al. 2009) and 2654 distinct human mature miRNAs available in miRbase (https://www.mirbase.org/) so far supports the idea that miRNAs are involved in complex regulatory networks. Their role has been established in several pathophysiologies including cancer, autoimmune, and metabolic disorders (Garo and Murugaiyan 2016; Naveed et al. 2017). However, in the past ten years, deep sequencing and miRNA microarray technology have evidenced that during the onset of pathogenic infection the host miRNAs regulate cellular responses (Bruscella et al. 2017; Zhou et al. 2018) resulting in signaling and physiological modifications. Parasites are known to have complex interactions with their specific hosts and these interactions are becoming a leading research field for infectious diseases. miRNAs have been involved in both the inflammatory response during the induction of the immune response and the modulation of innate and adaptive immune responses in infectious diseases (Pockar et al. 2019). Moreover, miRNAs also participate in mediating intercellular communications as they are secreted into vesicles or circulating extracellular fluids as exosomes, emphasizing its potential role as biomarkers for a variety of disorders, including parasitic diseases (Makarova et al. 2016). Although studies have highlighted the important role of miRNAs as regulators of gene expression related to the pathogenesis of numerous human diseases the specific functions of miRNAs in human parasitic infections are still not very clear. Thus, unraveling the regulatory roles of miRNAs in host−parasite interactions will not only provide new insights into our understanding of parasite disease pathogenesis but will also offer a foundation for new therapeutic approaches to be established. In this review, we demonstrate our current understanding of the influence of miRNAs in the development and progression of common parasitic diseases in humans, such as Malaria, Leishmaniasis, Amoebiasis, Chagas disease, Schistosomiasis, Toxoplasmosis, Cryptosporidiosis, Clonorchiasis, and Echinococcosis, as well as their possible use as clinically meaningful diagnostic biomarkers.

## miRNAs: fine modulators of parasitic infections

### Malaria and miRNAs

**Fig. 2:**
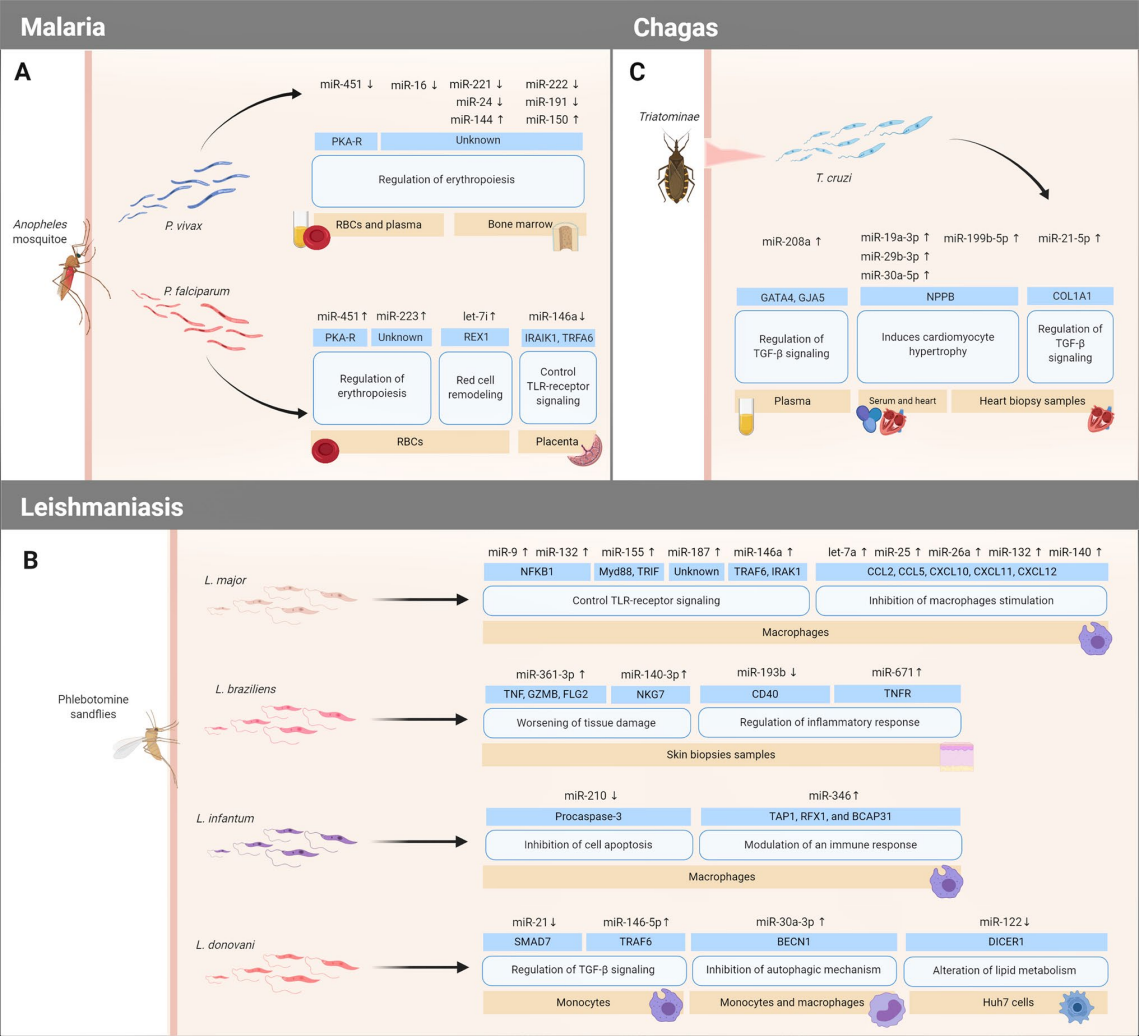
A graphical illustration of human microRNAs and their targets profile during host−parasite interaction of the most important insect vector transmitted parasitic diseases discussed in this review: (**a**) Malaria (**b**) Leishmaniasis, and (**c**) Chagas disease

*Plasmodium* is a genus of protist parasites that is transmitted by female *Anopheles* mosquitoes which injects sporozoites to vertebrate hosts (including humans) and quickly invades liver cells undergoing rapid multiplication causing malaria (Fig. 2a) (White 2017). There are six known *Plasmodium* species that infect humans, such as *P. vivax*, *P. ovale curtisi*, *P. ovale wallikeri*, *P. malariae*, *P. knowlesi,* and *P. falciparum* (Singh et al. 2017), and among them, *P. falciparum* is considered the deadliest since it is responsible for driving the most severe forms of the disease (Phillips et al. 2017; Garrido-Cárdenas et al. 2019). Malaria is one of the foremost infectious illnesses in the world (generally in low-income countries) affecting around 228 million people per year (World Health Organization 2019). Although it is widely known that *P. falciparum* lacks the classical functional RNAi machinery, accumulating evidence point to the possible exploitation of the host RNAi machinery or by employing a novel mechanism unique to *Plasmodium*, to manipulate host miRNA expression favoring their growth and survival leading to a potential alteration in the expression of erythrocytic miRNAs (Rathjen et al. 2006; Hakimi and Ménard 2010). It has been shown that *Plasmodium* parasite principally upregulates those host miRNAs whose target proteins are involved in immune response and downregulates those miRNAs that participate in the inhibition of parasitic translation, host cell proliferation, metabolism, and survival (Table 1) suggesting a high probability to be involved in the manipulation of both MAPK/ERK (Paroo et al. 2009) and Transforming Growth Factor-β (TGF-β) signaling pathways (Lourembam et al. 2013). Increasing evidence suggests that miRNA-451, miR-223, and let-7i are significantly upregulated in *Plasmodium*-infected red blood cells (RBCs) (Xue et al. 2008; Lamonte et al. 2013) targeting genes, such as Protein Kinase A Regulatory (PKA-R) and Reduced Expression protein 1 (REX1) involved in the regulation of erythropoiesis (the process of RBCs production) and red cells remodeling (Lamonte et al. 2013). Moreover, few studies indicated that host miRNAs may participate in the prognosis of the disease after the *Plasmodium* infection, supporting the idea that they could be considered as biomarkers for the diagnosis of parasite−host response and the disease progression. For example, miR-146a rs2910164 polymorphism, which could affect the expression level of mature miR-146a (downregulation), has been associated with increased susceptibility to *P. falciparum* infection in placental samples of pregnant women (Van Loon et al. 2019). Taganov and colleagues (2006) demonstrated that miR-146a is involved in the regulation of important immune response genes, such as tumor necrosis factor receptor-associated factor 6 (TRAF6) and IL-1 receptor-associated kinase 1 genes (IRAK1). On the other hand, *P. vivax* infection triggers a downregulation of miR-451 and miR-16 in plasma and RBCs of malaria patients and those have been also involved in the regulation of erythropoiesis by targeting PKA-R (Chamnanchanunt et al. 2015). In addition, it has been demonstrated that miR-221, miR-222, miR-24, and miR-191 were downregulated in the bone marrow of *P. vivax* infected patients, while miR-144, which is generally upregulated during erythropoiesis, and miR-150, which drives megakaryocyte formation while inhibiting erythropoiesis, were overexpressed, respectively (Baro et al. 2017). To date, a few reports have been reviewed to understand the consequence of *Plasmodium* infection on the host's miRNA expression profile and the disturbance of cellular homeostasis (Shrivastava and Rajasubramaniam 2018), however, the specific interaction between human miRNAs and malaria infection is still unknown.

**Table 1:** Relevant miRNAs involved in different parasitic diseases^a^

Disease	Parasite	miRNAs	Target genes	Biological mechanism	Sources	Ref
Malaria	*P. falciparum*	miR-451 ↑	PKA-R	Regulation of erythropoiesis	RBCs	(Xue et al. 2008; LaMonte et al. 2013; Chamnanchanunt et al. 2015)
		miR-223 ↑	Unknown			
		let-7i ↑	REX1	Red cell remodeling		
		miR-146a ↓	IRAK1 and TRAF6	Control TLR-receptor signaling	Placental samples	(Van Loon et al. 2019; Taganov et al. 2006)
	*P. vivax*	miR-451 ↓	PKA-R	Regulation of erythropoiesis	RBCs and plasma	(Chamnanchanunt et al. 2015)
		miR-16 ↓	Unknown			
		miR-221 ↓			Bone marrow	(Baro et al. 2017)
		miR-222 ↓				
		miR-24 ↓				
		miR-191 ↓				
		miR-144 ↑				
		miR-150 ↑				
Leishmaniasis	*L. major*	miR-9 ↑	NFKB1	Control TLR-receptor signaling	Macrophages	(Guerfali et al. 2008; Bazzoni et al. 2009; Lemaire et al. 2013)
		miR-132 ↑				
		miR-155 ↑	Myd88 and TRIF			
		miR-187 ↑	Unknown			
		miR-146a ↑	TRAF6, IRAK1			
		let-7a ↑	CCL2, CCL5, CXCL10, CXCL11, and CXCL12	Inhibits macrophages stimulation		
		miR-25 ↑				
		miR-26a ↑				
		miR-132 ↑				
		miR-140 ↑				
	*L. braziliens*	miR-361-3p ↑	TNF, GZMB, and FLG2	Worsening of tissue damage	Skin biopsies sample	(Lago et al. 2018)
		miR-140-3p ↑	NKG7			
		miR-193b ↓	CD40	Regulation of inflammatory response		(Nunes et al. 2018)
		miR-671 ↑	TNFR			
	*L. infantum*	miR-210 ↓	Procaspase-3	Inhibition of cell apoptosis	Macrophages	(Lemaire et al. 2013)
		miR-346 ↑	TAP1, RFX1, and BCAP31	Modulation of an immune response		(Diotavelli et al. 2018)
	*L.donovani*	miR-21 ↓	SMAD7	Regulation of TGF-β signaling	Monocytes	(Geraci et al. 2015)
		miR-146b-5p ↑	TRAF6			
		miR-30a-3p ↑	BECN1	Inhibition of autophagic mechanism	Monocytes and macrophages	(Singh et al. 2016)
		miR-122 ↓	DICER1	Alteration of lipid metabolism	Huh7 cells	(Ghosh et al. 2013)
Amoebiasis	*E. hystolytica*	miR-526b-5p ↑	XIAP, BAK1, BNIP3L	Induces cell cycle arrest, tumor suppression, and apoptosis	Epithelial colon cells	(Lopez-Rosas et al. 2019)
		miR-643 ↑	XIAP, BCL2L1, BCL2L10, BCL2L11, BCL2L14, API5			
		miR-615-5p ↑	BCL2L1, BCLAF1, BMF, AIFM3, AATK			
		miR-525 ↑	BCL2L1, BLC2L13, BAG3, BAG1, XIAP, CASP7, BMF			
		miR-150 ↑	BCL2L2, NAIF1, CASP7, CASP8, AIFM3, AIFM2, CFLAR			
		miR-409-3p ↓	BCL2, BCL2L11, BCL2L15			
Chagas Disease	*T.cruzi*	miR-208a ↑	GATA4 and GJA5	Regulation of TGF-β signaling	Plasma	(Linhares-Lacerda et al. 2018)
		miR-19a-3p ↑	NPPB	Induces cardiomyocyte hypertrophy	Serum and heart biopsy sample	(Nonaka et al. 2019)
		miR-29b-3p ↑				
		miR-30a-5p ↑				
		miR-199b-5p ↑			Heart biopsy sample	
		miR-21-5p ↑	COL1A1	Regulation of TGF-β signaling		
Schistosomiasis	*S. japonicum*	miR-150-5p ↑	KANK4, DRD1, and MT1H	Actin reorganization and cell contractility	Liver sample	(Cabantous et al. 2017)
		miR-146b-5p ↑	Unknown	Regulation of lipid metabolism		
		miR-143-3p ↑	PL1N1 and VNN1	Induces late fibrosis		
		miR-199a-3p ↑		Accumulation of triglycerides		
		miR-10a-5p ↑	KANK4 and VNN1	Regulation of TGF-β signaling		
		miR-4521 ↑	CTNNA3	Reorganization of extracellular matrix		
		miR-31-5p ↑	Unknown	Live fibrosis progression		
		miR-222-3p ↑	KANK4 and SLC39A8			
		miR-221-3p ↑	DRD1			
		miR-663b ↓	GBP5	Oxidative stress damage		
		Bantam*	Unknown	Unknown	Serum	(Meningher et al. 2016)
		miR-2c-3p*				
		miR-3488*				
		miR-2a-5p*				
Toxoplasmosis	*T. gondii*	miR-125 ↑	Unknown	Inhibition of apoptosis	Macrophages	(Cai et al. 2013, 2014)
		miR-27b ↑				
		miR-30c ↑				
		miR19a ↑	BIM			
		miR-19b ↑				
		miR-20a ↑				
		miR-17 ~ 92 cluster ↑			HFF	(Zeiner et al. 2010)
		miR-106b ~ 25 cluster ↑	Unknown			
		miR-146a ↑	IRAK1 and TRAF6	Control TLR-receptor signaling	HFF-Me49 infected cells	(Cannella et al. 2014)
		miR-146a ↓			HHF-RH infected cells	
		miR-132 ↑	APAF1, KRAS, MAPK3, and PPP2R5E	Regulation of cell apoptosis and immune response	Neuroepithelial cells	(Ngô et al. 2017; Xiao et al. 2014)
Cryptosporidiosis	*C. parvum*	let-7 family ↓	SNAP23 and SOCS4	Activation of TLR4/ NF-kB signaling	Biliary epithelial cells	(Hu et al. 2010, 2013)
		miR-27b ↑	KSRP			(Zhou et al. 2012)
		miR-98 ↓	SOCS4	Regulation of cytokine signaling		(Hu et al. 2010; Sato et al. 2017)
		miR-34b-5p ↓	ELAVL1, RAB10, RAB14	Regulation of cell apoptosis and immune response	HCT-8 cells	(Wang et al. 2019)
		miR-3591-3p ↓	ELK4, SOS2, TAB2, DAXX, FGF14, MAPK3			
		miR-18b-3p ↓	Unknown			
		miR-3976 ↓				
Clonorchiasis	*C. sinensis*	miR373 ↑	MMP9	Modulation of cell adhesion, migration, invasion, and metastasis	HuCCT1 cells and H69 cells	(Pak et al. 2014)
		miR24 ↑	PTP			
		miR342-5p ↑	AKT1	Activation of proinflammatory mediators		
		miR181d ↑	CDH13 and RASSF1	Inactivation of tumor suppressor genes via hypermethylation		
		miR31 ↑	LAST2 and PPP2R2A	Inhibition of cancer prevention pathways		
		miR185 ↑	PTEN and PTPN13			
		miR136 ↑	Unknown	Cell proliferation and inhibition of tumor suppression		
		miR-95 ↑				
		miR-93 ↑				
		miR-153 ↑				
		miR-16-2 ↑				
		miR-195 ↑		Suppression of cell growth		
		miR-199a-3p ↑	CAV2	Increases the proliferative and survival cell activities		
		let7i ↓	TLR4, RAS, MYC, and HMGA2	Inhibition of proliferation and differentiation of tumor cells		
		let7a ↓				
		miR-124 ↓	STAT3 and EZH2	Inhibition of cell proliferation. Induction of apoptosis, and suppression of tumor growth	HuCCT1, H69, HepG-2, and gastric cancer cells	
Echinococcosis	*E. granulosus*	let-7g-5p ↑	IL-13, IL-10, and IL-6	Proliferation and activation of macrophages, inflammation, apoptosis and oxidative damage	Whole blood samples	(Mariconti et al. 2019)
		let-7a-5p ↑				
		miR-26a-5p ↑	TMEM184B	Increases the expression of type I IFN		
		miR-26b-5p ↑	PTEN	Modulation of NF-κB pathway		
		miR-195-5p ↑	BCL2	Promotion of apoptosis		
		miR-16-5p ↑				
		miR-30c-5p ↑	Unknown	Regulation of the innate immunity, type I IFN signaling		
		miR-223-3p ↑	GZMB and HDAC2			
		egr-miR-71*	Unknown	Unknown	Plasma	(Alizadeh et al. 2020)
		egr-let-7*				
		miR-19↓	COL1A1 and COL3A	Suppression of cell proliferation by blocking signal transmission in the TGF-β pathway	LX-2 cells and liver tissue	(Zhang et al. 2016)
	*E. multilocularis*	miR-483-3p ↑	LBR	Promotes wound healing and cancer progression	Plasma	(Ren et al. 2019)

*Parasite derived miRNAs

^↑^Indicates upregulated

^↓^Indicates downregulated

### Leishmaniasis and miRNAs

The genus *Leishmania* was first described in 1903 for the highly pathogenic species *L. donovani*, but since then several pathogenic species of the genus *Leishmania* have been reported. *Leishmania* flagellates are transmitted to vertebrates by the bite of infected female phlebotomine sandflies generating leishmaniasis with symptoms ranging from skin lesions to fatal leishmaniasis (Fig. 2b) (Akhoundi et al. 2017; Borghi et al. 2017; Derici et al. 2018). It has been estimated up to 1 million new cases every year, resulting in 26,000–65,000 deaths worldwide (World Health Organization 2020). Over the past decade, miRNAs have been shown to be related to the pathogenesis of leishmaniasis (Table 1). It has been seen that at the first hours of the *L. major* infection upregulation of miR9, miR132, miR-155, miR-187, and miR-146a occur which is related with the control of TLR-receptor signaling and targeting transcripts, such as Nuclear Factor NF-κ-B p105 (NFKB1), Myeloid differentiation primary response 88 (Myd88), TIR domain-containing adaptor protein-inducing Interferon β (TRIF), TRAF6, and IRAK1 in macrophages, suggesting that these miRNAs are negative regulators of fine-tuned inflammatory reactions. Likewise, an upregulation of let-7a, miR-25, miR-26a, miR-132, and miR-140 in *L. major*-infected human macrophages led to a negative correlation on the expression of their specific chemokine targets CCL2, CCL5, CXCL10, CXCL11, and CXCL12 inhibiting macrophage stimulation (Guerfali et al. 2008; Bazzoni et al. 2009; Lemaire et al. 2013). On the other hand, it has been stated that miR-361-3p and miR-140-3p were significantly overexpressed in cutaneous leishmaniasis lesions (CL) generated by *L. braziliensis* infection as compared to normal skin samples targeting genes involved in worsening of tissue damage such as TNF, Granzyme B (GZMB), Filaggrin-2 (FLG2) and Natural Killer cell Granule protein 7 (NKG7) (Lago et al. 2018). While downregulation of miR-193b and upregulation of miR-671 are correlated with their respective target genes CD40 and TNF receptors (TNFR) modulating the inflammatory response in lesions caused by this parasite (Nunes et al. 2018). In addition, Lemaire and colleagues (2013) highlighted the downregulation of miR-210 during *L. infantum* infection which led to inhibition of cell apoptosis by targeting procaspase-3 in monocyte-derived macrophages. Likewise, Diotavelli et al. (2018) described the upregulation of miR-346 during *L. infantum* infection of human U937 and THP-1-derived macrophages decreasing the mRNA level of major histocompatibility complex- or interferon-associated genes, such as antigen peptide transporter 1 (TAP1), regulatory factor X1 (RFX1) and B-cell receptor-associated protein 31 (BCAP31) involved in both immune response regulation and cell survival under endoplasmic reticulum stress during infection; thus, miR-346 could be considered as an enticing target for anti-*Leishmania* approaches. Ghosh et al. (2013) assessed an interesting connection between altered lipid metabolism during *L. donovani* infection and Huh7 cells miR-122 levels (downregulation) by targeting DICER1. Moreover, Geraci and colleagues (2015) demonstrated significant correlations between miR-21 (downregulation) and miR-146b-5p (upregulation) in *L. donovani* infected dendritic cells and specific members of the TGF-β signaling pathway SMAD7 and TRAF6. Furthermore, a decrease infectivity of *L. donovani* due to the inhibition of the autophagic mechanism via negative regulation of Beclin 1 (BECN1) in THP-1 and human monocyte-derived macrophages has been reported to be triggered by the upregulation of miR-30a-3p during infection (Singh et al. 2016).

### Amoebiasis and miRNAs

*Entamoeba histolytica* is a single-celled anaerobic protozoan parasite that causes human amoebiasis. It is spread by fecal–oral transmission and is most prevalent in areas plagued by overcrowding, poverty, and poor sanitation (Nourollahpour Shiadeh et al. 2016; Pineda and Perdomo 2017; Deere et al. 2019). Amoebiasis affects around 50 million people globally of which approximately 10% of infected individuals are at risk of contracting invasive amoebiasis that includes amoebic colitis and amoebic liver abscesses. Invasive amoebiasis kills up to 100,000 people worldwide annually, mainly in tropical countries (Saidin et al. 2019). The effects of parasites on host miRNAs expression have been described in a few species of protozoans and nematodes, but very little is known about *E. histolytica*. The contribution of miRNAs in amoebiasis has been described to act in the modulation of gene expression of physiological and pathophysiological factors (Table 1). It has been suggested that miRNA-controlled pathways such as clathrin receptor-mediated internalization of lipid and protein molecules, as well as gene regulation and signal transduction of the Ras family GTPase are disrupted during *E. hystolytica* infection (Mar-Aguilar et al. 2013). Interestingly, evidence indicates that the components of miRNA biogenesis machinery, such as Argonaute (AGO) are present in *E. histolytica* while Dicer protein is still elusive, suggesting the presence of some unknown mechanisms to regulate gene expression without a Dicer enzyme (Mar-Aguilar et al. 2013). However, recent studies have shown an important dysregulation of miRNAs repertoire in epithelial colon cells, specifically, López-Rosas and coworkers (2019) evidenced that after 45 min of *E. histolytica* infection, there is an upregulation of miR-526b-5p, miR-643, miR-615-5p, miR-525, and miR150, and a downregulation of miR-409-3p, which altogether may impact in the expression of genes involved in at least five major biochemical pathways in SW-480 cells, including biosynthesis of unsaturated fatty acids, phosphatidylinositol 3-kinase/Protein kinase B (PI3K/AKT) signaling pathway, ubiquitin-mediated proteolysis, mRNA surveillance pathways, and apoptosis. Interestingly, the amoeba can induce apoptosis in host cells by partially altering miRNAs that regulate genes involved in lipid metabolism, the PI3K/AKT signaling pathway, and apoptosis. Of particular note, the aforementioned six modulated microRNAs potentially target apoptosis-related genes. Thus, the expression of B Cell Lymphoma 2 (BCL-2) protein family, BCL2 Antagonist/Killer 1 (BAK1), BCL2 Interacting Protein 3 Like (BNIP3L), X-linked Inhibitor of Apoptosis Protein (XIAP), Apoptosis Inducing Factor Mitochondria associated 2 and 3(AIFM 2 and AIFM3), Apoptosis Inhibitor 5 (API5), BCL2 Associated transcription Factor 1 (BCLAF1), Apoptosis-Associated Tyrosine Kinase (AATK), BAG1, BAG3, BMF, Nuclear Apoptosis Inducing Factor 1 (NAIF1), CFLAR, and Caspase 7 and 8 (CASP7 and CASP8) can potentially be regulated by the aforesaid miRNAs suggesting their possible usage in the clinical diagnosis of amoebiasis (Lopez-Rosa et al. 2019).

### Chagas disease and miRNAs

Chagas disease (CD) is an anthropozoonosis caused by the protozoan parasite *Trypanosoma cruzi* (Fig. 2c) which is transmitted by insect vector Triatominae or kissing bugs and affects about 10–12 million people just in America leading to approximately 50,000 deaths per year (Corti and Villafañe 2017; Pérez-Molina and Molina 2018; De Oliveira et al. 2020). The infection has two successive phases. The acute phase is characterized by a high parasitemia, usually asymptomatic or oligosymptomatic, while it may progress to the chronic phase with neurological, cardiac, digestive, or cardiodigestive clinical complaints (Pérez-Molina and Molina 2018; De Souza 2019). Among chronic Chagas patients, modulation of gene expression in myocardial tissue is mostly associated with immune response, metabolism, and cell stress response (Ferreira 2014). Over the past decade, the central role of miRNAs has been established to impact the resistance to infection and the pathogenesis of CD (Table 1). Linhares-Lacerda et al. (2018) detected higher levels of miR-208a in plasma samples from patients with chronic CD and they suggested that this might be correlated with TGF-β stimulation and regulation of genes involved in cardiac hypertrophy and fibrosis, such as GATA binding 4 (GATA4) and Gap Junction Alpha-5 (GJA5). More recently, Nonaka et al. (2019) reported an elevated level of miR-19a-3p, miR-29b-3p, and miR-30a-5p in serum, and miR-19a-3p, miR-21-5p, miR-29b-3p, miR-30a-5p, and miR-199b-5p in heart samples from chronic CD patients suggesting that some of them might be correlated with cardiac injury and disease severity, targeting Natriuretic Peptide B (NPPB) and Collagen type I Alpha 1 chain (COL1A1). However, the relationship of miRNAs with CD pathogenesis could be further explored in order to validate new biomarkers or molecular targets for therapeutic intervention.

### Schistosomiasis and miRNAs

Schistosomiasis (also known as Bilharziasis) is a parasitic infection caused by several species of blood-flukes of the genus *Schistosoma* and it is one of the most prevalent zoonotic diseases, affecting over 258 million individuals in 54 countries (McManus et al. 2018; Chuah et al. 2019; Salari et al. 2020). Trematode parasites *S. mansoni* (mainly distributed in Africa, South America, Caribbean, and the Middle East), *S. haematobium* (Africa and the Middle East), and *S. japonicum* (China and Southeast Asia) are the main species that cause this human disease (Meningher et al. 2020). Infections take place in freshwater bodies, where schistosomes penetrate human skin; followed by penetration, schistosome cercariae migrate to the host portal−mesenteric vein system where the female worm lay a large number of eggs that are either discharged into the environment through feces or urine or are retained in host tissues where they induce inflammation (Colley et al. 2014; Wu et al. 2015). Several lines of evidence have been demonstrated that miRNAs can modulate schistosomiasis pathogenesis (Table 1). For example, Cabantous et al. (2017) showed that in the liver of *S. japonicum* infected patients the levels of miR-150-5p, miR-146b-5p, miR-143-3p, miR-199a-3p, miR-10a-5p, miR-4521, miR-31-5p, miR-222-3p, and miR-221-3p were elevated, while miR-663b was present in low level. Furthermore, they reported that the predicted target genes of the aforesaid miRNAs, such as KANK4, Dopamine Receptor D1 (DRD1), Metallothionein-1H (MT1H), PL1N1, Vanin 1 (VNN1), Catenin Alpha-3 (CTNNA3), SLC39A8, and Guanylate-Binding Protein 5 (GBP5) are involved in crucial processes implicated in hepatic fibrosis progressions, such as cellular proliferation and differentiation, reorganization of the extracellular matrix, lipolysis, and cellular detoxification. To date, four schistosomal miRNAs have been isolated from extracellular vesicles in sera from *Schistosoma* infected individuals, such as Bantam, miR-2c-3p, miR-3488, and miR-2a-5p suggesting that can be used both as a diagnostic tool for infection and to monitor treatment effectiveness (Meningher et al. 2016). More recently, the role of miRNAs in the pathogenesis of hepatic fibrosis in schistosomiasis caused by both *S. japonicum* and *S. mansoni* has been reviewed, highlighting their role in the regulation of antifibrosis and profibrosis mechanisms (Chen et al. 2019). Moreover, the recent advances in characterizing miRNA profiles in extracellular vesicles secreted by *Schistosoma* species have upstretched the possibility for validating more parasite-derived miRNAs as potential biomarkers for schistosomiasis detection (Cabantous et al. 2017).

### Toxoplasmosis and miRNAs

*Toxoplasma gondii*, one of the most common human parasites in the world, is a ubiquitous pathogen that is the causative agent of toxoplasmosis and can infect a large range of hosts including humans. The transmission begins with the ingestion of contaminated raw meat and then the parasite starts to infect as many cells as possible. Manifestations are highly variable, ranging from asymptomatic to severe, especially in cases of brain and eye infection (Parlog et al. 2015; Assolini et al. 2017). Nearly all infections are silent, and it has been shown that this parasite specifically modulates the expression of essential miRNAs in the host, altering their response to the infection (Table 1). The first study in this subject demonstrated that *Toxoplasma* effectors are responsible for the alterations in host miR-17 ~ 92 and miR-106b ~ 25 family expression that are upregulated after infection with RH toxoplasma strain in primary human foreskin fibroblasts (HFFs) (Zeiner et al. 2010). Similarly, Cai and colleagues (2013) found an increased expression of miR-20a, miR-125, miR-19a, miR-19b, miR-27b, and miR-30c in human macrophage at 6 h and 12 h postinfection. Interestingly, some of those miRNAs (miR-19a, miR-19b, and miR-20a) are part of the miR-17 ~ 92 cluster and have been associated with a novel mechanism for the regulation of apoptosis by inhibiting proapoptotic molecule BIM allowing *T. gondii* to evade immune responses through this mechanism (Cai et al. 2014). On the other hand, Cannella and colleagues (2014) used an infected HFF with two different *Toxoplasma* strains (Me49 and RH) and reported an upregulation of miR-146a in HFF infected with Me49 and a downregulation in the RH infected cells, targeting IRAK and TRAF which are fine modulator of TLR-receptor signaling pathway. Furthermore, it is also known that *T. gondii* affects the human brain in many pathways related to epilepsy, neurodegeneration, and cancer. A positive regulation of miR-132 has been reported in the human neuroepithelial cell line infected with RH-2F, PRU, and CTG strains which have been related to a modulation in the expression of genes involved in the metabolism of dopamine, such as apoptotic protease-activating F-factor 1 (APAF1), Kirsten Rat Sarcoma (KRAS), MAPK3 and PPP2R5E (Xiao et al. 2014; Ngô et al. 2017). Finally, it has been recently reviewed that this parasite also features its own miRNA processing system and possesses the capacity to secret exosomes that contain miRNAs (Menard et al. 2019). Changes in the miRNA profiles of the host in *T. gondii* infection represent a powerful mechanism for a better understanding of this pathology.

### Cryptosporidiosis and miRNAs

The genus *Cryptosporidium* is composed of protozoan parasites that infect the gastrointestinal epithelium and other mucosal surfaces of their host, including humans (Vanathy et al. 2017). Transmission occurs through the fecal−oral path, and sources of infection with *Cryptosporidium* include contaminated food or water (Dumaine et al. 2019). Human cryptosporidial infections have been related mainly to *C. parvum* and the severity of infection may range from an asymptomatic shedding of ingested oocysts to a serious life-threatening, and prolonged disease (Ryan et al. 2014; Lender et al. 2015). The exact molecular pathogenic mechanisms of cryptosporidial infection are still not completely known. However, a growing number of functional studies have documented the role of miRNAs in the response of human hosts to *Cryptosporidium* (Table 1). For example. increasing evidence suggests that host miRNAs help in the elimination of the parasites by regulating TLR4 and NF-κB signaling pathways and with the regulation of the release of antimicrobial peptides (Chen et al. 2007; Hu et al. 2013). Specifically, it has been demonstrated that *C. parvum* infection reduces the expression of the let-7 family miRNAs in biliary epithelial cells, which lead to an increase of synaptosome-associated protein 23 (SNAP23) expression, coordinating the subsequent release of exosomes carrying antimicrobial peptides (Hu et al. 2013). In addition, Zhou and colleagues (2012) demonstrated the role of miR-27b (upregulation) in the modulation of TLR4/NF-κB-mediated epithelial anti-*C. parvum* responses in human biliary epithelial cells, targeting KSRP. On the other hand, a couple of studies suggest that host miRNAs are also used by *C. parvum* to strengthen its own survival. Specifically, *C. parvum* infection downregulates the expression of miR-98 and let-7 family to induce the suppressor of cytokine signaling (SOCS4) proteins that are negative regulators of cytokine signaling (Hu et al. 2010; Sato et al. 2017). Recently, Wang and collaborators (2019) reported for the first time the miRNA expression profile of human intestinal epithelial cells infected with *C. parvum* and they evidenced that most miRNAs were not significantly differentially expressed in the infected HCT-8 cells as compared to uninfected cells. Nonetheless, they reported that the miR-34b-5p, miR-18b-3p, miR-3976, and miR-3591-3p were downregulated after *C. parvum* infection and those have been associated with the regulation of both apoptotic processes and epithelial immune responses, targeting genes, such as ELAVL1, RAB10, RAB14, ELK4, SOS2, TAB2, DAXX, fibroblast growth factor 14 (FGF14), and MAPK3.

### Clonorchiasis and miRNAs

Clonorchiasis is a food-borne parasitic disease caused by the fluke *Clonorchis sinensis* through the consumption of raw or undercooked freshwater fish, containing metacercariae of *C. sinensis*. Clonorchiasis generally appears as bile duct obstruction, biliary inflammation, liver cirrhosis, hepatic carcinoma, and cholangiocarcinoma (Wu et al. 2012; Tang et al. 2016). This parasite infects about 35 million people globally, mainly distributed in Asian countries, such as China, Japan, Vietnam, and Korea (Han et al. 2016). Although molecular mechanisms of carcinogenesis associated with hepatic fluke infestation are not completely understood, some studies have investigated variations in miRNA expression patterns and associations with specific biological functions in *C. sinensis* infection indicating a possible association between miRNA and cholangiocarcinogenesis (Table 1). Specifically, in vitro experiments using human cholangiocarcinoma cells (HuCCT1) treated with excretory-secretory protein (ESP) of *C. sinensis* have demonstrated an upregulation in expression levels of miR373, miR342-5p, miR-199a-3p, miR-195, miR185, miR181d, miR-153, miR136, miR-95, miR-93, miR31, miR24, and miR-16-2 along with the downregulation of miR-124a, let7i, and let7a, in a time-dependent manner compared with untreated controls. Similarly, ESP-treated normal cholangiocytes (H69) revealed that the expressions of nine miRNAs (miR-16-2, miR-93, miR-95, miR-153, miR-195, miR-199-3P, let7a, let7i, and miR-124a) were similarly regulated, showing that the cell proliferation and inhibition of tumor suppression mediated by these miRNAs are common to both cancerous and noncancerous cells (Pak et al. 2014). Functional clustering of these dysregulated miRNAs revealed their involvement in cell differentiation/proliferation, inflammation, metastasis, oncogene regulation, and DNA methylation by regulating various cancer-related signaling pathways, such as TGF-β, MAPK, TLR, and PI3K/AKT, and by targeting several genes, such as matrix metalloproteinase-9 (MMP9), Tyrosine-protein phosphatase (PTP), AKT serine/threonine kinase 1 (AKT1), CDH12M, Ras Association domain family member 1 (RASSF1), LAST2, PPP2R2A, Phosphatase and Tensin homolog (PTEN), PTPN13, Caveolin-2 (CAV2), TLR4, RAS, MYC, HMGA2, STAT3, and EZH2 (Pak et al. 2014; Yan et al. 2016). This information could allow the identification of potential targets and miRNA-associated genes involved in multiple oncogenic pathways during *C. sinensis* infection and establishing new tools that may function as indicators for the diagnosis and prognosis of the disease.

### Echinococcosis and miRNAs

Cystic echinococcosis (CE) and alveolar echinococcosis (AE), two severe zoonotic tapeworm diseases, are triggered by *Echinococcus granulosus* sensu lato (s.l.) and *Echinococcus multilocularis*, respectively (Wang et al. 2013; Wen et al. 2019). CE (considered as a neglected disease by the WHO) infects mainly the liver and lungs and it is categorized as a major public health problem resulting in 1.2 million cases per year (Mariconti et al. 2019; Alizadeh et al. 2020). On the other hand, AE, one of the most dangerous human parasitic zoonosis in the northern hemisphere, is mainly characterized by a tumor-like development of metacestodes in human livers causing around 200 cases per year (Geramizadeh and Baghernezhad 2016). MicroRNA-based diagnostics have attracted great interest in biomarker research for clinical diagnosis and monitoring of echinococcosis (Table 1). The recent studies show a substantial upregulation of eight miRNAs (let-7g-5p, let-7a-5p, miR-26a-5p, miR-26b-5p, miR-195-5p, miR-16-5p, miR-30c-5p, and miR-223-3p) in whole blood samples of patients with active larval cysts as compared to those with inactive cysts. These upregulated miRNAs have been involved in a variety of biological processes, such as macrophages proliferation and activation, inflammation, apoptosis, oxidative damage, targeting, modulation of the NF-κB pathway and type I interferon signaling by targeting genes, such as Interleukin 6, 10, and 13 (IL-6, IL-10, and IL-13), Transmembrane protein 184B (TMEM184B), PTEN, BCL2, GZMB, and Histone Deacetylase 2 (HDAC2) (Mariconti et al. 2019). Furthermore, Zhang and colleagues (2016) reported that the expression of miR-19 showed a significant reduction in hepatic stellate cells (LX-2 cells) treated with hydatid cyst fluid (HCF) in pericystic collagen-rich liver tissue of CE patients, as compared to normal liver, leading to a significant suppression of cell proliferation by blocking signal transmission in the TGF-β pathway, decreasing COL1A1 and COL3A protein expression rates, suggesting that miR-19 could be an important biomarker in humans infected with *E. granulosus* (s.l.). Likewise, Alizadeh and colleagues (2020) reported that egr-miR-71 and egr-let-7 can be detected in human plasma during hydatid cyst infection and can be used as possible biomarkers for the early diagnosis and monitoring of CE. Similarly, miR-483-3p that targets Lamin-B receptor (LBR) and has been associated with cancer progression, is assessed as a potential marker due to substantial upregulation in the plasma of AD patients as compared to normal controls that provide a new approach to the clinical diagnosis of AE (Ren et al. 2019).

## Conclusion

Identifying and characterizing parasite-specific miRNAs and their targets in hosts, as well as miRNAs interfering with host pathology, are crucial for a better understanding of the pathophysiology of parasitic diseases at the molecular level. The biological knowledge acquired about miRNAs, especially in biomedical research, is expected to be widely translated in the field of parasitology in the coming years since miRNAs have great potential to lead to a transition to a novel class of theranostic tool. Thus, we can expect the discovery of more and more specific miRNAs with highly specialized functions linked to cellular processes in parasites that can provide novel guidelines for the management of parasitic diseases.

## Data Availability

The figures were exported under a paid subscription. Created with BioRender.com.
